# Sensor-Based Motion Tracking System Evaluation for RULA in Assembly Task

**DOI:** 10.3390/s22228898

**Published:** 2022-11-17

**Authors:** Wenny Franciska Senjaya, Bernardo Nugroho Yahya, Seok-Lyong Lee

**Affiliations:** 1 Department of Industrial and Management Engineering, Hankuk University of Foreign Studies, Yongin 17035, Republic of Korea; 2 Faculty of Information Technology, Maranatha Christian University, Bandung 40164, Indonesia

**Keywords:** ergonomics, musculoskeletal disorders, risk assessment, wrist posture, multimodal sensors

## Abstract

Industries need a mechanism to monitor the workers’ safety and to prevent Work-related Musculoskeletal Disorders (WMSDs). The development of ergonomics assessment tools helps the industry evaluate workplace design and worker posture. Many studies proposed the automated ergonomics assessment method to replace the manual; however, it only focused on calculating body angle and assessing the wrist section manually. This study aims to (a) propose a wrist kinematics measurement based on unobtrusive sensors, (b) detect potential WMSDs related to wrist posture, and (c) compare the wrist posture of subjects while performing assembly tasks to achieve a comprehensive and personalized ergonomic assessment. The wrist posture measurement is combined with the body posture measurement to provide a comprehensive ergonomics assessment based on RULA. Data were collected from subjects who performed the assembly process to evaluate our method. We compared the risk score assessed by the ergonomist and the risk score generated by our method. All body segments achieved more than an 80% similarity score, enhancing the scores for wrist position and wrist twist by 6.8% and 0.3%, respectively. A hypothesis analysis was conducted to evaluate the difference across the subjects. The results indicate that every subject performs tasks differently and has different potential risks regarding wrist posture.

## 1. Introduction

Work-related Musculoskeletal Disorders (WMSDs) are the major outcome of occupational injuries in the industry [1]. The Korean Ministry of Employment and Labor reported 9440 cases of WMSDs in 2019; these case rates increased by 40.6% from the previous year [2]. WMSDs can decrease workers’ productivity and increase workers’ compensation costs as WMSDs are the main cause of absenteeism, lost working days, and temporary or permanent disability [3,4]. The dominant factors that lead to WMSDs in manual assembly tasks are awkward working postures, repetitive movements, and workloads [5,6,7]. Repetitive movements are a significant risk factor for upper limb WMSDs, especially for the arm and wrist. Some injuries and disorders affect the wrist, such as carpal tunnel syndrome, wrist tendonitis, and lateral epicondylitis [8].

Many studies have proposed a method to help industries evaluate their work environment. There is a trend in measuring technical ergonomics based on sensors, such as wearable inertial sensors [9,10]. Many ergonomic assessment research works rely on a vision-based approach utilizing either image [11,12] or motion sensors like Kinect [13] to focus on high-level activities (e.g., lifting, sitting, kneeling, hammering, and drilling) without considering the Therbligs activities. The most active body part in the assembly process is the hand (i.e., arm, wrist, and fingers). Therbligs motions (e.g., grasp, assemble, inspect, etc.) can be used to describe the low-level activity done by hand, and these motions are related to wrist kinematics [14]. For example, when a worker grasps something, the wrist might be extended or twisted. Existing works have addressed ergonomic assessment with low-level or Therbligs activities through two different approaches: manual assessment and biosensor-based assessment. The former approach mainly utilizes the body skeleton data to determine some body parts’ position scores automatically; however, the wrist twist and position score were determined manually by the ergonomist [15,16]. Manual assessment is time-consuming [12] and is mostly based on the ergonomist’s assumptions. The latter approach uses a biosensor-based assessment (e.g., Shimmer wearable sensors, Delsys TrignoTM, Electrodes) to measure the wrist kinematics [17,18]; however, the sensor attached to the worker’s body may cause discomfort and affect postural behavior [13]. Work using unobtrusive sensors was addressed by [19] with two limitations in wrist kinematics measurement: weak joint points selection and missing the adjustment score. The weak joint points selection mostly produced a perpendicular angle and resulted in a high-risk score. In addition, the previous work did not cover the adjustment score, which is important for measuring the risk of wrist posture.

Accurately measuring the risk posture is essential because the measurement results can be used as analysis material for the evaluation of the workplace setup and workers’ training to reduce the risk in order to prevent WMSDs and maintain safety at the workplace. In addition, posture risk assessment can also be used for advanced applications, such as virtual training and monitoring systems. Many industrial projects adopt virtual training to increase productivity during training and solve the problem of the lack of training instructions. It is essential that a training session accurately simulates an actual working environment and any challenges that a worker might experience in the workplace. Monitoring systems in the industry can help workers to raise awareness of occupational health and safety by providing training on safety and risk assessments for high-risk jobs (e.g., hazard inspection at a construction site) [20].

This study focuses on developing a method to measure wrist kinematics based on unobtrusive sensors. Wrist kinematics occurs as the result of complex 3D movements between individual carpal bones, including between carpal bones and the bones surrounding the forearm [21]. To evaluate the overall posture risk in assembly tasks, we perform RULA and synchronize multimodal unobtrusive sensors, a body tracking sensor, and a hand tracking sensor. Both sensors are unobtrusive; therefore, the sensors do not interfere or change the postural behavior of workers. Recent studies have proven that multimodal sensor fusion can achieve high-performance generalization and tackle some problems that are too complex to solve with single sensor values [22]. In addition, we not only measure a certain posture from an activity but also evaluate the transition movement. Therefore, the proposed method can evaluate and detect potential postures that cause WMSDs in workers’ movements more precisely. Unlike previous works that measured the potential risk based on the frequency of the RULA grand score [19], this study attempts to elaborate the adjustment of wrist kinematics (i.e., flexion–extension and radial–ulnar deviation) and forearm rotation (i.e., pronation–supination) to enhance the ergonomics assessment method. The main contributions of our works are summarized as follows:

- We propose a geometric-based wrist kinematics measurement for radial–ulnar deviation and wrist twist measurement based on the flexion–extension angle and forearm pronation–supination from the hand tracking data sensor.
- We present a comprehensive ergonomics assessment (i.e., RULA) using a derived wrist posture measure, along with a body posture measure, in the assembly process automatically.
- We present an extensive experiment to show a personalized ergonomic assessment using multimodal unobtrusive sensors (i.e., body tracking and hand tracking sensors).

The remaining sections of this work are structured as follows: the second section presents a summary of the related works in the fields of ergonomics assessment and wrist kinematics. The third section describes our proposed method. The fourth section describes the experimental settings and evaluation results. The fifth section discusses the findings from the experimental results. The last section concludes this study and the potential future works from this study.

## 2. Related Work

### 2.1. Ergonomics Assessment

Ergonomics assessment is the method to evaluate the body posture of workers while they are accomplishing a task. This assessment helps evaluate workplace design, reduce the risk of WMSDs, and improve worker productivity [23]. Most previous studies have used RULA and REBA to assess ergonomics; both methods use joint angle measurements to determine the risk level of body posture. The joint angle is measured for every body part, such as the upper arm, lower arm, trunk, wrist, etc. One of the most challenging parts to measure is the wrist. Some previous studies have an ergonomist manually set the wrist score based on an image or video [13,15,16]. A possible reason for this is that they only utilized data from one source to measure the body joint angle and lacked data to measure the wrist. Other previous studies determined the wrist score automatically; however, they did not explain the wrist joint angle measurement in detail.

The previous studies usually used OpenPose to detect poses from images [13,15,16] or motion capture technology to record body movements [24,25,26]. Some previous studies addressed motion capture and motion tracking using diverse types of sensors, such as an accelerometer shirt [27], inertial sensors, a smartphone, or a camera. Some of the previous studies used recorded sensor data to perform pose-by-pose reconstructions. In [28], the author proposed reconstructing a virtual character’s full-body movement method for different movement behaviors (e.g., walking, running, etc.) by using a single IMU. The authors in [29] proposed a Gaussian Process Latent Variable Model (GPLVM) to visualize high dimensional data. GPLVM was used by [30] to represent pose data with latent coordinate points and used a Hidden Markov Model (HMM) to segment the phase. The phase at each frame is automatically determined based on the HMM classifier. A Multilayer Perceptron (MLP) model has been applied to define the mapping between the training sensor data and the latent coordinates points [30]. The author in [31] presented the iNEMO inertial measurement unit to estimate the orientation of human body segments. iNEMO consists of various Micro Electro-Mechanical Systems (MEMS) that can estimate the orientation of the human body segment [31].

This study uses the data from the multimodal unobstructive sensors to provide the ergonomics assessment based on RULA. Furthermore, previous studies have only evaluated several postures, while this study evaluates the whole posture across a sequence of activities that occur while assembling a drawer. Table 1 shows the list of related works that developed the ergonomics assessment method. Previous studies usually used OpenPose to detect poses from images [15,16,32] or used motion capture technology to record body movements [24,25]. This study uses data from multimodal unobstructive sensors to provide the ergonomics assessment based on RULA. 

### 2.2. Wrist Kinematics

Some previous studies focused on the wrist assessment for a specific activity, such as laparoscopic surgery [36,37] and activity during mouse use [38]. The authors in [36] measured the flexion–extension angles of the surgeon’s wrist using wireless electro-goniometer sensors and CAPTIV version L7000 software. The sensors were placed on the back of the right and left hands and forearms [36]. Ref. [37] utilized five sensors for monitoring the forces exerted by the distal phalanges of the surgeon’s thumb; index, middle, and ring fingers; and palm. All data were examined with custom-made software [37]. Ref. [38] used an infrared three-dimensional (3D) motion analysis system (Optotrak Certus, Northern Digital, Ontario, Canada) to measure the posture of the upper extremities; moreover, six-degrees-of-freedom force–torque sensors (ATI, Apex, NC, USA) were attached to each arm support plate and the mouse pad. This study evaluated six support conditions (i.e., forearm support, flat palm support, raised palm support, forearm + flat palm support, forearm + raised palm support, and no support) and the joint angles were calculated from the Euler angles; these are defined by the rotation matrices that describe the orientation of the distal segment relative to the proximal segment concerning the anatomical position, and the vertical position was used to determine the joint angles [38].

Some previous studies estimated wrist kinematics by collecting hand motion data using a biosensor. The author in [18] proposed a novel regression scheme for supervised domain adaptation (SDA), wherein the domain shift effects were effectively reduced to enhance the inter-subject performances of CNN in the wrist kinematics estimation. This study recorded sEMG signals using the Delsys TrignoTM system. The electrodes were placed over five primary wrist muscles in the right forearm: Flexor Carpi Radialis (FCR), Flexor Carpi Ulnaris (FCU), Extensor Carpi Radialis Longus (ECRL), Extensor Carpi Radialis Brevis (ECRB), and Extensor Carpi Ulnaris (ECU) [18]. Another previous work proposed a Convolutional Neural Network (CNN)-Long Short-Term Memory (LSTM) hybrid model to fully explore the temporal–spatial information from surface electromyography (sEMG). The wrist movements were simultaneously recorded with Shimmer wearable sensors, which were attached to the back of the testing hand [17].

## 3. Method

**Figure 1:**
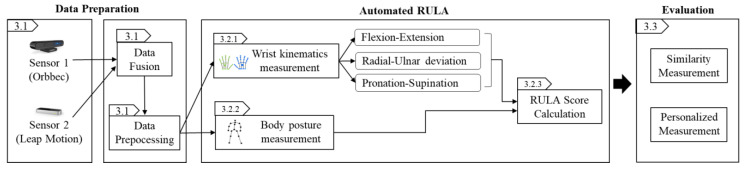
Workflow of Ergonomics Assessment.

Figure 1 shows our ergonomics assessment workflow, which consists of three sections: data preparation, automated RULA, and evaluation. Data preparation has three phases: data collection, data fusion, and data processing. In the automated RULA section, the process starts with the expert labeling the data with a RULA score. The expert assesses the worker’s posture by visualizing the data using 3D visualization tools and matches it with the video recorded during data collection. We use the expert-labeled data as ground truth. The next phase is the proposed ergonomics assessment method that evaluates the body posture and wrist kinematics (i.e., flexion–extension, radial–ulnar deviation, and pronation–supination) to determine the RULA score. The last section is the evaluation, wherein we measure the similarity of the RULA scores that were calculated by the expert and automatically calculated by the system. In addition, to evaluate the heterogeneity between subjects, we perform hypothesis testing to justify the personalized measurement.

### 3.1. Data Preparation

The data was collected from 12 subjects—eleven subjects were right-handed and one subject was left-handed. All subjects included in the data collection signed the “Research Subject Confidentiality Agreement” form. We collected the data using a body tracking sensor and a hand tracking sensor. The first sensor is the Orbbec camera, which has a sampling rate of 30Hz and is used to record body movements. The second sensor, the Leap Motion, aims to record hand motions with a 64 Hz sampling rate. Since both sensors have different sampling rates, we have to balance the sampling rates to fuse the data from both sensors. We used Piecewise Aggregate Approximation (PAA) to reduce the dimensionality of the time series to 20 Hz.

PAA uses three steps to reduce dimensionality. First, it divides the original time series into N equal-length segments. Second, it computes the mean of each segment. Third, it replaces the value of the segment with its mean. Given this, $XT=\left[XT_{1},XT_{2},\ldots ,XT_{k},\ldots ,XT_{m}\right]$ is a multivariate time series where m denotes the number of variables $\left(1\leq k\leq m\right)$; $XT_{k}$ is an n-length univariate time series, where $XT_{k}=\left\{xt_{1},xt_{2},\ldots ,xt_{i},\ldots ,xt_{n}\right\}$. $xt_{i}\left(1\leq i\leq n\right)$ i denotes the value in time; and *n* is the entire length of the time series $XT_{k}$. For example, $XT_{1}=\left\{xt_{1},xt_{2},\ldots ,\;xt_{i},\ldots ,xt_{n}\right\}$ is a univariate time series with *n*-length, and PAA approximates time series $XT_{1}$ into $\bar{XT_{1}}=\left\{\bar{xt_{1}},\;\bar{xt_{2}},\ldots ,{\bar{xt}}_{i},\ldots ,\;\bar{xt_{N}}\right\}$, where *N* is the number of segments [39]. Equation (1) shows the formula ${\bar{xt}}_{i}$ for calculating each ${\bar{xt}}_{i}$.

(1) $${\bar{xt}}_{i}=\frac{N}{n}\sum_{j=\frac{n}{N\left(i-1\right)}+1}^{\left(\frac{n}{N}\right)i}xt_{j}$$

Using Ref. [39], we adjusted the data with a 20 Hz sampling rate for the next step. During data collection, we also label the data with three labels that represent the activity levels: high-level, middle-level, and low-level activities.The data are labeled after the dimension reduction process is complete. Furthermore, because RULA requires calculating the joint angles for six body sections, we cannot skip the calculation for any section; therefore, we remove all null value rows.

### 3.2. Labeling by Expert

**Figure 2:**
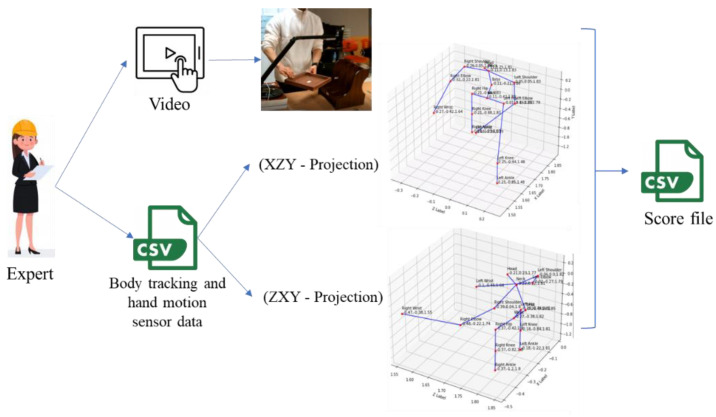
Annotation used by the expert.

We validate our automated RULA score calculation using an expert’s evaluation. The expert herein means a specialist who understands the whole assembly process, follows the data collection process and has knowledge of ergonomics assessment and risk posture. The expert also has a background in industrial and computer science. The expert-labeled data includes the RULA score and risk level. Since labeling postures for 12 subjects will take a long time, the expert only evaluates and labels the posture for every ten rows of data (per 0.5 s). The posture evaluation is based on 3D visualizations and recorded videos. The 3D visualization is provided through two types of projection: a front-right view (XZY-projection) and back-left view (ZXY-projection). The total number of expert-labeled postures labeled is 4729. The expert assesses the left upper arm, right upper arm, left lower arm, right lower arm, right wrist, left wrist, neck, trunk, and legs for every pose. We develop a visualization tool to make labeling easier. This tool displays a 3D visualization of the skeleton with the XZY-projection or ZXY-projection. Figure 2 describes the annotation process that the expert uses.

### 3.3. Automated RULA

#### 3.3.1. RULA Score Calculation

The RULA score is determined based on the body joint angle at each region. Six body regions need to be evaluated: the upper arm, lower arm, wrist, neck, trunk, and legs. There are several aspects that must be considered in the evaluation of each part of the body. For example, the right upper arm evaluation involves the right upper arm position, right shoulder raise, and right upper arm abduction [40]. The first step in determining the upper arm position score is the calculation of the joint angles between the right elbow, right shoulder, and right hip. Afterward, we can determine the upper arm position score according to the RULA worksheet threshold.

RULA divides the body assessment into two sections: section A and section B. Section A consists of the arm and wrist, while section B consists of the neck, trunk, and legs [40]. Both sections generate an intermediate RULA score of A and B, respectively. Afterward, the RULA grand score is calculated by combining both intermediate RULA scores [16]. In this study, intermediate RULA scores A combines the multiple sensor angle calculation. The joint angle of the upper arm and lower arm position is calculated using the data from the body tracking sensor, while the wrist position is calculated using the data from the hand tracking sensor.

**Figure 3:**
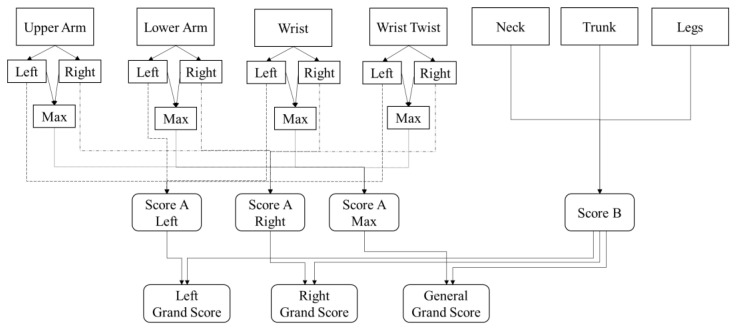
RULA Scoring Mechanism.

We calculated the joint angle from the left and right sides. Furthermore, we calculated a Max score that is defined by the highest score between the left and right position. To determine intermediate RULA score B, we need the score from the neck position, trunk position, and legs. The joint angle of the neck and trunk position are calculated using the data from the body tracking sensor, while the legs score is directly defined (i.e., 2) for all postures given that all of the subjects perform all of the tasks in a standing position, and the legs and feet are not supported. After scores A and B are calculated, we can calculate the grand score (score C). There are three kinds of grand score: the left grand score, which is calculated based on score A from the left side and score B; the right grand score, which is calculated based on score A from the right side and score B; and the general grand score, which is calculated based on the Max score and score B. Figure 3 illustrates the RULA scoring mechanism. The RULA grand score is categorized into four risk levels: negligible, low, medium, and high. Table 2 presents the risk levels with the actions that should be taken for each risk level.

#### 3.3.2. Wrist Kinematics Measurement

This section discusses wrist kinematics measurement. The wrist bone structure consists of eight carpal bones that connect proximally to the forearm’s radius and connect distally to the hand’s five metacarpals. The flexion–extension and radial–ulnar deviation of the wrist are its global motions. The wrist is able to perform those movements due to articulation at the radiocarpal joint, midcarpal joint, the carpo-metacarpal joint, and between individual carpal bones. The forearm pronation–supination motion is possible due to the distal radioulnar joint [21].

**Figure 4:**
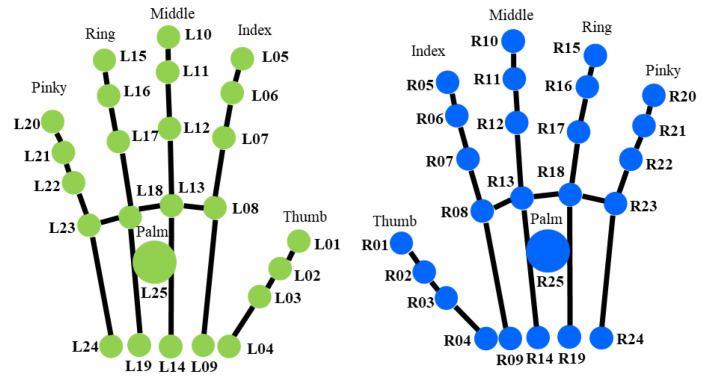
Finger joints.

RULA involves the evaluation of the wrist flexion–extension, wrist radial–ulnar deviation, and forearm pronation–supination to assess the wrist section. This study assesses the wrist section with the data collected by the finger tracking sensor. Figure 4 shows the finger joints from the finger tracking sensor. Each hand has 25 joints, and all of the joints are represented by a 3D coordinate (x,y,z). The finger joints are used to calculate the wrist position and wrist twist score. As shown in Figure 4, we label every finger joint—‘L’ for the left hand and ‘R’ for the right hand. A list of the finger joints is provided in Table A1.

Table 3 shows the finger joints that are involved in wrist kinematics measurement. Four wrist kinematics need to be measured: wrist flexion–extension, wrist radial–ulnar deviation, forearm pronation–supination, and the range of the forearm when pronating and supinating.

1. Wrist flexion–extension

**Figure 5:**
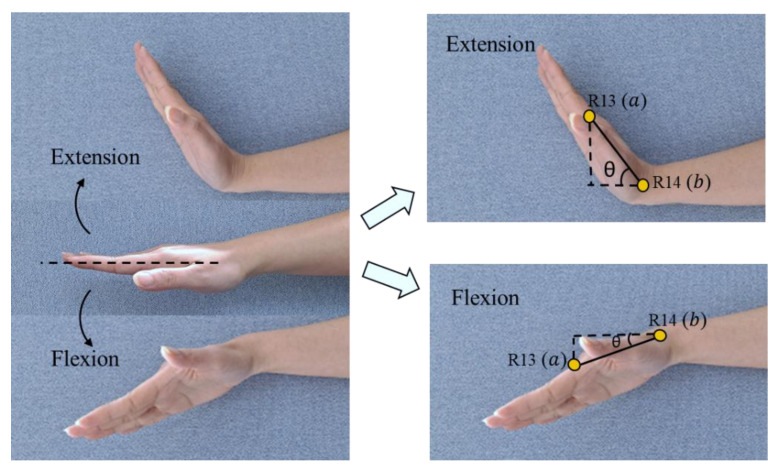
Calculate wrist twist position (flexion–extension).

The calculation of the wrist’s flexion–extension angle involves two 3D points from the middle finger. Figure 5 illustrates the point position from the right hand, where R13 and R14 represent the middle finger proximal phalanges endpoint and middle finger metacarpals endpoint, respectively.

In this case, $a\left(x_{1},\;y_{1},z_{1}\right)$ is the first point and $b\left(x_{2},\;y_{2},z_{2}\right)$ is the second point. Equation (2) shows the formula that is used to calculate the angle of wrist position [41].

(2) $$cos\theta =\frac{a\cdot b}{\left|a\right|\left|b\right|}$$

2. Wrist radial–ulnar deviation

**Figure 6:**
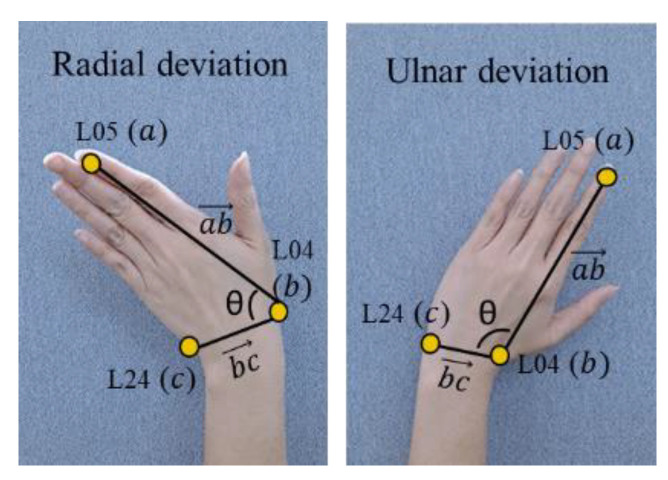
Wrist bending position (radial–ulnar deviation).

We also assess the wrist radial–ulnar deviation (wrist bending) to adjust the RULA score for the wrist. We assume that if the wrist is bent 15° or more, the wrist score will increase by 1. The joint involved in assessing wrist bending is (R05, R04, R24) for the right hand and (L05, L04, L24) for the left hand. The three joint points represent the position of the start point of the index finger of the distal phalanges, the metacarpals endpoint of the thumb, and the metacarpals endpoint of the pinky finger. The illustration of the assessment of the wrist’s radial–ulnar deviation angle is shown in Figure 6.

The deviation angle is the angle between two vectors that is formed by three joint points, where each joint consists of 3D coordinates (x,y,z). In this case, $\;a\left(x_{1},\;y_{1},z_{1}\right)$, $b\left(x_{2},y_{2},z_{2}\right)$, and $c\left(x_{3},y_{3},z_{3}\right)$ represent the first, second, and third joint positions, respectively. Equation (3) shows the formula that is used to calculate the joint angle.

(3) $$\cos\theta =\frac{\vec{ab}\cdot \vec{bc}}{\left|\vec{ab}\right|\left|\vec{bc}\right|}$$

3. Forearm pronation–supination

**Figure 7:**
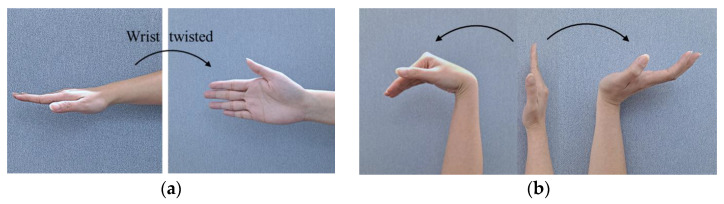
Wrist twist assessment: (**a**) wrist is twisted in mid-range (pronation–supination); (**b**) wrist is at or near the end of range.

The next position that should be assessed is the pronation–supination (wrist twist). Pronation is the rotation of the hand from the handshake position to the position where the palm is facing downward. In contrast, supination is the rotation of the hand from the handshake position to an upward-facing palm surface position [40]. Figure 7 illustrates wrist twists in mid-range (a) and the wrist at or near the end of range (b). To evaluate the wrist twist, we calculate the angle between two normal vectors. $\vec{IPR_{nv}}$, $\vec{CPR_{nv}}$, $\vec{IPL_{nv}}$, and $\vec{CPL_{nv}}$ represent the normal vectors of the initial position of the right hand, the current position of the right hand, the initial position of the left hand, and the current position of the left hand, respectively.

The normal vector is a function that is used to find the normal vector from the coronal plane. Suppose $a\left(x_{1},\;y_{1},z_{1}\right)$, $b\left(x_{2},y_{2},z_{2}\right)$, and $c\left(x_{3},y_{3},z_{3}\right)$ represent three joint positions, then the equation that is used to find the normal vector is defined as shown in Equation (4) [41].

(4) $$NV=\left(b-a\right)\times \left(c-a\right)$$

**Figure 8:**
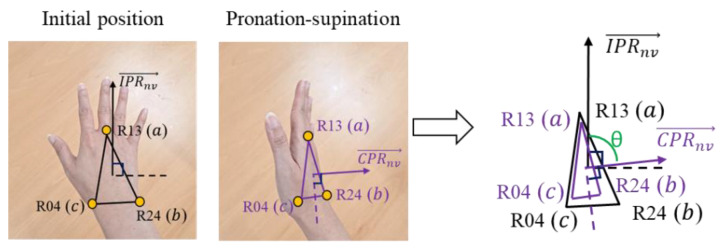
Wrist twist (pronation–supination) angle calculation.

Joints L13, L04, and L24 are involved in finding the normal vector for the left hand, while the normal vector for the right hand is defined by joints R13, R04, and R24. Figure 8 is an illustration of the wrist twist (pronation–supination) angle calculation. The angle between two planes is calculated to evaluate the pronation–supination position. The angle is determined between two normal vectors from two planes. In this case, the two normal vectors are determined by the initial position ($\vec{IPR_{nv}}$ and $\vec{IPL_{nv}}$) and the current position ($\vec{CPR_{nv}}$ and $\vec{CPL_{nv}}$).

**Figure 9:**
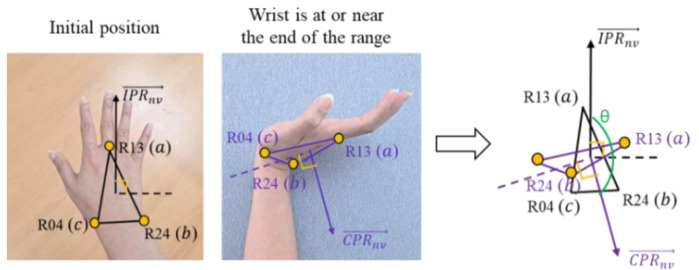
Wrist twist position in the range.

For adjusting the wrist twist posture assessment, Figure 9 illustrates the evaluation of the wrist twist posture in the flexion–extension range. The angle calculation for this assessment is also used for the calculation between the two normal vectors that are produced from the initial position plane and the current position plane. Equations (5) and (6) define the formula that is used to calculate the angle of the wrist twist for the right and left hand, respectively.

(5) $$\mathrm{Right}\;\mathrm{Hand}:\;\;\cos\theta =\frac{\vec{IPR_{nv}}\cdot \vec{CPR_{nv}}}{\left|\vec{IPR_{nv}}\right|\left|CPR_{nv}\right|}$$

(6) $$\mathrm{Left}\;\mathrm{Hand}:\;\;\cos\theta =\frac{\vec{IPL_{nv}}\cdot \vec{CPL_{nv}}}{\left|\vec{IPL_{nv}}\right|\left|CPl_{nv}\right|}$$

#### 3.3.3. Body Posture Measurement

**Figure 10:**
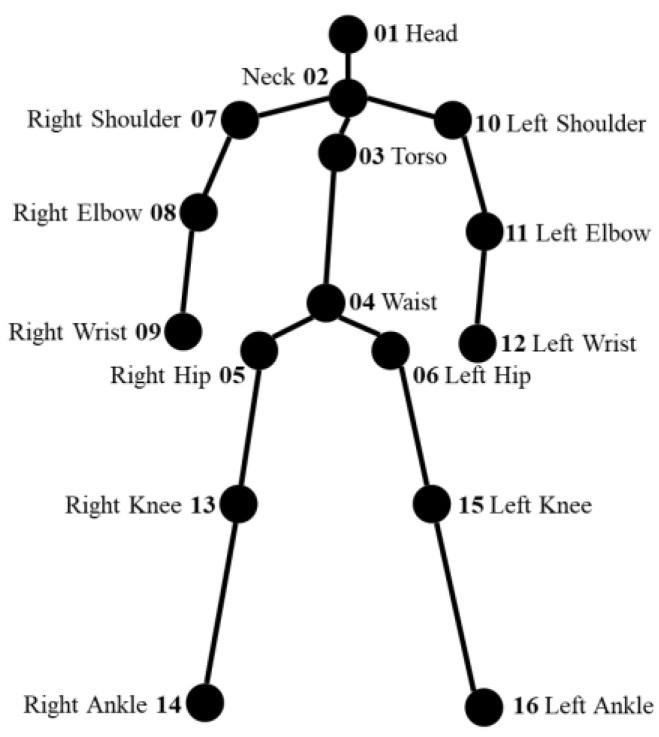
Body joints.

The measurement method for body posture (i.e., upper arm, lower arm, neck, trunk, and legs) is discussed in this section. Figure 10 shows the body joints from the body tracking sensor. There are 16 body joints, and each joint is represented by a 3D coordinate $\left(x,y,z\right)$. A list of the body joints is provided in Table A2. The body joints are used to calculate the score for the upper arm, lower arm, neck, and trunk.

Table 4 shows the body joints that are involved in angle calculations. Since $a\left(x_{1},\;y_{1},z_{1}\right)$, $b\left(x_{2},y_{2},z_{2}\right)$, and $c\left(x_{3},y_{3},z_{3}\right)$ represent the first, second, and third joint positions, respectively; the calculation of the joint angle is performed by Equation (2). Since the subjects are working in standing positions without any support during the assembly process, the legs score is determined to be 2. Furthermore, we do not consider the neck twist, muscle, or force score.

The *NV*() is a function that is used to find the normal vector from the coronal plane. The equation that is used to find the normal vector is defined as shown in Equation (4) [41].

### 3.4. Evaluation

To evaluate the proposed method, this study compares the score from expert observation with that calculated by our method; moreover, we measure the similarity of the RULA score on each high-level activity. Since $ES=es_{1},es_{2},\ldots ,es_{n}$ is the list of scores from expert observation and $SS=ss_{1},\;ss_{2},\;\ldots ,\;ss_{n}$ is the list of the scores from system calculation, Equation (7) shows the similarity $numSim\left(es,ss\right)$ between two numbers, es and ss. Then, Equation (8) is the formula that is used to calculate the mean similarity $tsim\left(ES,SS\right)$. The intervals for the similarity measurement are [0, 1], where 1 indicates the maximum similarity [42].

(7) $$numSim\left(es,ss\right)=1-\frac{\left|es-ss\right|}{\left|es\right|+\left|ss\right|}$$

(8) $$tsim\left(ES,SS\right)=\frac{1}{n}\overset{n}{\underset{a=1}{\sum }}numSim\left(es_{i},ss_{i}\right)$$

Furthermore, a hypothesis analysis was also conducted to evaluate and compare the differences in wrist posture across the subjects. This analysis was performed using a one-way ANOVA and *t*-test.

## 4. Experiment and Results

### 4.1. Laboratory Setup

**Figure 11:**
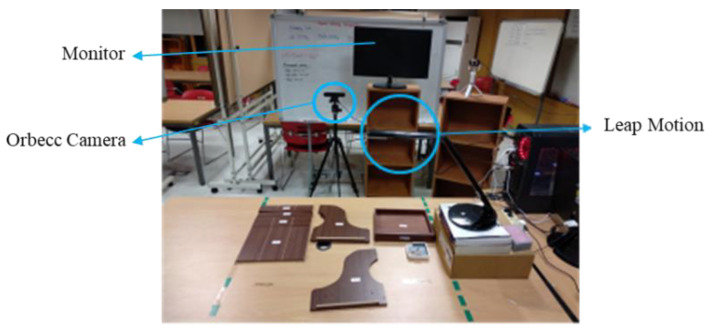
Laboratory Setup.

The data were collected from 12 subjects. Every subject had to follow the assembly steps that were given by the instructor. Figure 11 shows the laboratory setup during data collection. We used two sensors to record the assembly activity. The Orbecc Astra Pro camera collected body movement data from the subjects during assembly; it was placed in front of the subjects. The second sensor was Leap Motion, which was used to collect finger movement during the assembly process. It was located on the table, and we ensured that the sensor could cover the entire assembly area. There was also a monitor that showed the skeleton movements from the Orbecc and Leap Motion sensors. This laboratory setup was also used for training sessions by the subjects to train in the assembly process before the data collection process.

**Figure 12:**
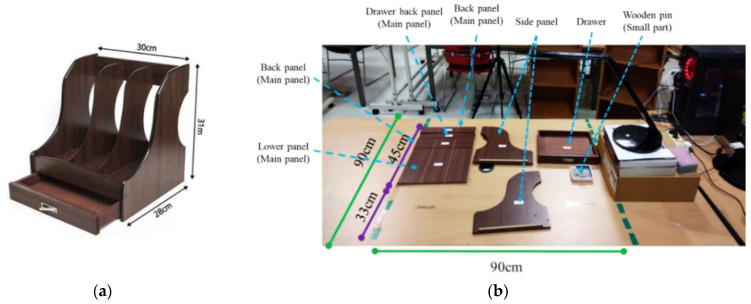
(**a**) Product; (**b**) Panel positioning for assembly.

Figure 12 shows the product we assembled (a) and the panel positioning we used during the data collection (b). The size of the product is 30 × 28 × 31, which consists of main panels, side panels, wooden pins, and a drawer. The height of the table is 72 cm. We collected the data from 12 subjects with a height range of 155–178 cm. The average subject’s height was 170 cm. Each subject assembled the product three times, and we chose the best performance based on the number of null values during the assembly process.

**Figure 13:**
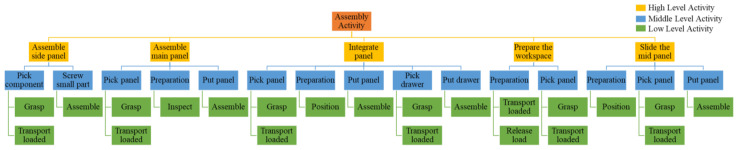
List of Assembly Activity.

The process for drawer assembly is divided into five main steps: assemble side panel, assemble main panel, integrate panel, prepare the workspace, and slide the mid-panel. Figure 13 shows the list of assembly activities with three levels of activity. The low level is represented by the Therblig activity. Therblig is a system for assessing the motions required to complete a task, and it is able to examine the smallest of motions. There are 18 motions that can be used to describe the task [43,44]. This study only uses the six Therbligs that are the most relevant to our assembly task (i.e., grasp, transport loaded, assemble, inspect, position, and release load). The middle-level label aims to bridge the low-level activities and high-level activities.

**Figure 14:**
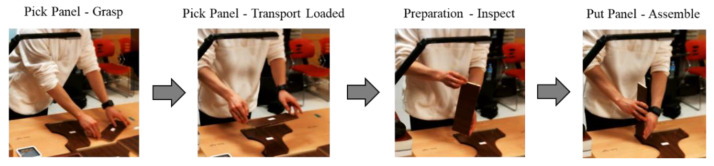
The example of activities to assemble the main panel.

Figure 14 shows an example of one of the activities in the assembly process in our data. The “assemble the main panel” activity consists of three steps. The first step is to pick the panel. The “pick panel” activity consists of two activities: “Grasp” and “Transport Loaded”. The second step is the “preparation” activity, which involves inspecting the panel position; therefore, the panel is ready to be installed on the main panel. The last step is to assemble the main panel.

### 4.2. Evaluation Results

This section conveys two different subsections to show the results of our experiments through two lenses: similarity measurement and personalized measurement. The similarity measurement takes place based on the respective body parts. The personalized measurement considers the statistical hypothesis in order to justify that the proposed measurement could be utilized as a personalized measure.

#### 4.2.1. Similarity Measurement

The evaluation of proposed method is evaluated by measuring the similarities between the scores calculated by the expert and the scores calculated by the system. The evaluation is divided for each high-level activity (i.e., assemble side panel, assemble main panel, integrate panel, prepare the workspace, and slide the mid-panel) and is based on the mechanism in Figure 3. We divided the evaluation results into five tables (Table 5, Table 6, Table 7, Table 8 and Table 9).

In the first table (Table 5), we present the similarities between the section A (i.e., upper arm, lower arm, wrist, and wrist twist) scores for the left and right sides. The similarity score for the left side is higher than that for the right side. A possible reason for this is the right hand is more active than the left hand; therefore, the left hand is more likely to stay in the same position.

The second table (Table 6) shows the similarities for the section A scores when we use the highest score between the right or left score (Max score) for the upper arm, lower arm, and wrist. As a comparison with the previous study, we measured the similarities between the wrist position and wrist twist score, which were calculated by the previous study method [19], then compared it with the similarities score from the current study method. The similarities results can be seen in the third table (Table 7). Beforehand, the previous study had not performed a score comparison analysis and only considered the higher score (Max score) from the two sides. Other than that, the previous study did not address the adjustment score (i.e., radial–ulnar deviation and range of pronation–supination) and instead relied on the ergonomist’s evaluation. Based on the results, the current study enhances the performance of the ergonomics assessment; it improves the similarity scores for the wrist position and wrist twist by 6.8% and 0.3%, respectively. The slight difference in scores for wrist twist is due to the score on wrist twist being measured as only 1 or 2. Table 1 shows the previous studies related to ergonomics assessment; however, most of them only focused on body assessments and the wrist score was manually assessed by an expert (ergonomists). Moreover, some previous studies did not explain their wrist assessment. Therefore, we can only compare our proposed method with one previous study [19]. We also had difficulty implementing our method with another dataset because no public dataset provided a RULA score.

The fourth table (Table 8) presents the evaluation for section B (i.e., neck, trunk, and legs). Due to all of the subjects performing all of the tasks in the standing position, the legs score is directly defined (i.e., 2) for all postures. Therefore, we only present the similarities results for the neck and trunk. The “integrate panel” activity has the lowest similarity. A possible reason for this is that the “integrate panel” activity is a difficult task to perform, and each subject performs the task differently. Compared to our previous study, the current study has an 82% average similarity score for the neck, while the method in our previous study was only 77.2%; this means that changing the torso joint point to the waist joint point can improve the ergonomic assessment performance.

The last table (Table 9) presents the similarities in the RULA grand score. In the experiment, we calculated three kinds of RULA grand scores. First, the Right RULA grand score is calculated based on the score in section B and the right-side score from section A. Second, the Left RULA grand score is calculated from the section B score and the left-side score of section A. Third, considering that the higher the RULA score the higher the risk level, we calculated the General RULA grand score from section B and the Max score from section A using the mechanism in Figure 3. Based on the results, the general grand scores are higher than the right and left sides.

#### 4.2.2. Personalized Measurement

This section evaluates the significant differences among the RULA of all subjects with a one-way ANOVA and *t*-test. The null hypothesis is H_0_: µ_1_ = µ_2_ = µ_3_ = … = µ_s_, where *s* is the number of subjects and α = 0.05. The *p*-value for the ANOVA result is 1.1078 × 10^−41^, which shows that H_0_ is rejected. This result justifies that there is at least one subject that is different from other subjects. For a more in-depth analysis, we performed a *t*-test on every pair of subjects.

Table 10 and Table 11 present the *t*-test results for subjects 1–12. Most of the results are significant, which means that there are differences between subjects in performing the tasks; this shows that even though the subject follows the instructions and has a training session, every subject performs the tasks in their own way. The underlined numbers are the non-significant differences results, which means that they were similarly performed assembly tasks.

## 5. Discussion

**Figure 15:**
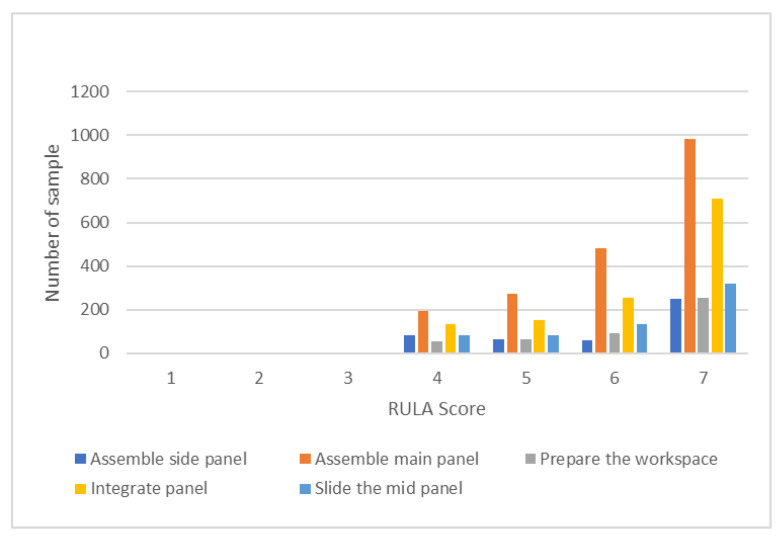
RULA score distribution.

In this section, we discuss the results of the evaluation methods based on the high-level activities. Figure 15 shows the distribution of the RULA grand scores from each high-level activity. Each activity has a different number of samples. Table 12 indicates the number of samples from the 12 subjects, wherein the RULA grand scores were generated using this system.

Based on the similarity measurement results, the overall similarity scores achieve more than 80% similarity. The high similarity scores for wrist position (i.e., flexion–extension and radial–ulnar deviation) and wrist twist (i.e., pronation–supination and the adjustment posture) indicate that our proposed method can measure wrist kinematics and detect potential risk factors; moreover, it improved the performance of ergonomics assessment similarity scores for wrist position and wrist twist by 6.8% and 0.3% on average.

**Figure 16:**
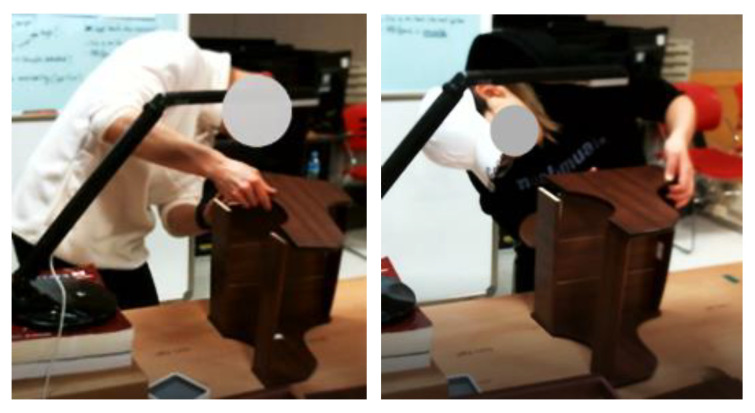
Installation of the side panel to the main panel.

Regarding the high-level activities, the activity “Assemble side panel” has the highest average score. This activity consists of simple tasks, such as attaching the wooden pin to the side panel. It can be seen from Figure 15 that this activity has the fewest high-risk RULA scores. On the contrary, the “Integrate panel” activity has the lowest average score. Possible reasons for this could be that this activity involves a difficult task. This can be seen from Figure 15, which indicates that the activity has the highest number of high-risk RULA scores; moreover, the Orbbec sensor cannot properly capture the joint data because the view is blocked by the drawer when the subject attaches a side panel to the main panel, especially considering the data for the arm position (see Figure 16). A RULA score is determined from the scores from the six body regions. The score for each body region is determined based on the joint angle. The joint angle is calculated based on the position of the body joints that is represented by a 3D coordinate (*x, y, z*). The 3D coordinate for each body joint is detected and recorded by the Orbbec sensor. Therefore, if the Orbbec sensor cannot properly capture the joint position data, it will affect the evaluation results. Furthermore, the difference in how workers install panels can also affect the result—i.e., sometimes the sensor view for the left hand is blocked, and sometimes the right hand is blocked. The Orbbec sensor actually works well for tracking body motions. Furthermore, since the Orbbec sensor is an unobtrusive sensor, workers can do their tasks without being disturbed by sensors being attached to their bodies; however, if there is a possibility of obstruction, it might be better to use more than one sensor and place it on the other side.

Based on the statistical hypothesis testing, it showed that there is a significant difference among most of the subjects (refer to Table 10 and Table 11). Some possible factors that impact this are handedness and work duration. In terms of handedness, some subjects claimed to be left-handed. For example, subject 5 and subject 6 had different dominant hands (subject 5 was left-handed while subject 6 was right-handed) and showed significant differences in the statistical hypothesis—although they have similar heights (170 and 168 cm) and have almost the same work duration (4.33 and 4.28 min). In terms of work duration, subject 9 and 10 showed a significant difference. Although both subjects have similar heights (170 and 166 cm, respectively) and both are right-handed, subject 9 was the subject with the fastest working time (3.63 min), while subject 10 had the longest working time (6.48 min); this can indicate that significant differences occur due to the handedness and work duration.

Some results showed an insignificant difference. For example, subject 5 and subject 11 had insignificant differences in their results, which means there was no difference in their risk. A possible reason for this is that both subjects are left-handed, which can indicate similar work patterns. A similar work duration can possibly cause an insignificant difference. For example, subject 4 and subject 5 have similar work durations—4.32 and 4.33 min, respectively.

The risk assessment method in this study can help industries evaluate workplace design to make workers work more efficiently. Furthermore, the method can also provide evaluation methods and suggestions to workers related to their posture in order to increase health awareness and prevent injuries.

In our experiment, the calculation of the RULA score is separate from data collection. We first collected the data from the 12 subjects, then combined all the data. During data collection, the joint can be detected and recorded in real time. We also cleaned the data before calculating the RULA score. As explained in Section 3.3.1, the calculation of the RULA score starts from the joint angle calculation for every body section and is used to determine the score based on the joint angle. Afterward, the calculation process continues with the calculation of the intermediate score (RULA score of section A and section B). The last step is the calculation of the RULA grand score, then saving all of the calculation results.

The total number of expert-labeled postures is 4729. After we measured the similarity between the RULA scores calculated by the expert and the scores calculated by the system, we generated the score for all poses from the 12 subjects. The total number of poses is 54,321. Our risk assessment method required around 0.043156 s to calculate the RULA grand score. This indicates that our method can be implemented for an ergonomics assessment system to evaluate poses in real time. The details of the execution time can be seen in Table A3.

Our method focuses on the development of ergonomics assessments for monitor the safety of workers and the prevention of WMSDs; however, other related domains that use ergonomics assessments, such as elderly health monitoring, worker efficiency management, and medical training, can also benefit from applying our proposed method.

A limitation of this study is that the evaluation of the method only used laboratory data. The muscle and force scores were not considered because the sensors only collected data on the joint positions. A comparison with the existing method is limited, given that no previous studies adopted a combination of body angle calculations and wrist posture assessments. Furthermore, due to the different devices used to collect the data, there is a dissimilarity in data features from the previous studies.

## 6. Conclusions

This study proposed a geometric-based wrist kinematics measurement to detect potential risk factors related to wrist position and wrist twist. Our study is able to provide an ergonomics assessment that combines wrist kinematics measurements with body posture measurements during an assembly process based on multimodal unobtrusive sensors (i.e., body tracking and hand tracking sensors). An extensive experiment and evaluation are provided to evaluate our proposed method. The evaluation results show that the current study improves the performance of the ergonomics assessment for wrist position and wrist twist. In addition, the personalized measurement results show that every subject is unique, and even though the subject has a training session before data collection, each subject still has differences from other subjects; this shows that humans perform the same task in their own way and have different potential risks.

In future research, we plan to evaluate our method by extracting data from a real-world industrial environment, analyze the sensitivity of the RULA score changes in similar postures with a slight difference in joint angle, and compare them with other ergonomics assessment tools. In addition, we also plan to build a warning system that provides an alert to a worker if they’re in a high-risk posture. This system can be used for assessing training, especially with new workers.

## Figures and Tables

**Table 1:** List of Related Works.

Ref.	Data	Body Joint	Finger Joint	Assessment Tools	Wrist Score
[15]	Image	17	-	RULA	Set manually
[16]	Image and video	25	-	RULA	Set manually
[3]	Image	17	-	RULA	Set manually
[11]	Image	-	-	RULA	Not available
[33]	Skeleton	25	-	RULA	Set manually
[13]	Skeleton	12	-	RULA	Set manually
[34]	Image	26	-	RULA	Not available
[32]	Image	17	-	REBA	Set manually
[35]	3D Model	22	-	RULA and REBA	Not available
[10]	Survey and wearable sensors	4	-	RULA	Not available
[24]	Skeleton	25	-	EAWS	Not available
[9]	Wearable inertial sensors	17	-	RULA and REBA	Not available
Ours	- Body-Tracking Sensor - Hand-Tracking Sensor	16	50	RULA	Calculate by system

**Table 2:** RULA Risk level.

Score	Risk Level	Action to Be Taken
1–2	Negligible	Acceptable posture if it is not repeated for a longer period
3–4	Low	Further investigation and change may be needed in future
5–6	Medium	The investigation and change are required soon
7	High	The investigation and change are required immediately

**Table 3:** RULA wrist posture scoring criteria.

Wrist Posture		Wrist Kinematics	Side	Formula	Score
Wrist	Wrist Position	flexion–extension	Right	∠R13, R14	+1 (0°) +2 (15° up, 15° down) +3 (>15° up, >15° down)
			Left	∠L13, L14	
	Wrist is bent from midline	radial–ulnar deviation	Right	∠R05, R04, R24	+1 (15° left, 15° right)
			Left	∠L05, L04, L24	
Wrist Twist	Wrist is twisted in mid-range	pronation–supination	Right	$∠\;\vec{IPR_{nv}}$, $\vec{CPR_{nv}}$	+1 (75°,105°)
			Left	$∠\;\vec{IPL_{nv}}$, $\vec{CPL_{nv}}$	
	Wrist is at or near the end of the range	range of pronation–supination	Right	$∠\;\vec{IPR_{nv}}$, $\vec{CPR_{nv}}$	+2 (105°, 165°)
			Left	$∠\;\vec{IPL_{nv}}$, $\vec{CPL_{nv}}$	

**Table 4:** RULA body posture score criteria.

Body Regions		Side	Formula	Score
Upper Arm	Upper arm position	Right	∠08, 07, 05	+1 (−20°, 20°) +2 (−∞, −20°) +2 (20°, 45°) +3 (45°, 90°) +4 (90°, ∞)
		Left	∠11, 10, 06	
	Shoulder is raised	Right	∠04, 02, 07	+1 (90°, ∞)
		Left	∠04, 19, 02	
	Upper arm is abducted	Right	∠02, 07, 08	+1 (20°, ∞)
		Left	∠02, 10, 11	
Lower Arm	Lower arm position	Right	∠09, 08, 07	+1 (60°, 100°) +2 (0°, 60°) +2 (100°, ∞)
		Left	∠12, 11, 10	
	Arm is working across the midline	Right	∠07, 03, 09	+1 (90°, ∞)
		Left	∠10, 03, 12	
	Arm is out to the side of the body	Right	∠08, 07, 05	+1 (30°, ∞)
		Left	∠11, 10, 06	
Neck	Neck position		∠01, 02, 04	+1 (0°, 10°) +2 (10°, 20°) +3 (20°, ∞) +4 (−∞, 0°)
	Neck is side bending		90 - (∠10, 02, 01)	+1 (20°, ∞)
Trunk	Trunk position		180 - (∠01, 04, [0,0,1])	+1 (0°) +2 (0°, 20°) +3 (20°, 60°) +4 (60°, ∞)
	Trunk is twisted		∠04, 06, *NV* (02, 05,06)	+1 (20°, ∞) to left and right
	Trunk is side bending	Right	∠02, 04, 05	+1 (20°, ∞)
		Left	∠02, 04, 06	

**Table 5:** Similarity measurement results for section A (right and left).

High-Level Activity	Upper Arm		Lower Arm		Wrist Position		Wrist Twist		
	Right	Left	Right	Left	Right	Left	Right	Left	AVG
Assemble side panel	0.891	0.902	0.914	0.953	0.94	0.947	0.846	0.916	0.914
Assemble main panel	0.87	0.873	0.853	0.861	0.941	0.942	0.971	0.837	0.894
Prepare the workspace	0.882	0.914	0.917	0.882	0.934	0.939	0.837	0.89	0.899
Integrate panel	0.831	0.873	0.839	0.822	0.954	0.947	0.821	0.895	0.873
Slide the mid-panel	0.865	0.909	0.876	0.856	0.944	0.951	0.833	0.907	0.893
AVG	0.87	0.89	0.88	0.87	0.94	0.95	0.86	0.89	

**Table 6:** Similarity measurement results for section A (Max score).

High-Level Activity	Upper Arm	Lower Arm	Wrist Position	Wrist Twist	AVG
Assemble side panel	0.926	0.865	0.979	0.961	0.933
Assemble main panel	0.897	0.845	0.971	0.968	0.920
Prepare the workspace	0.881	0.832	0.963	0.949	0.906
Integrate panel	0.899	0.824	0.981	0.966	0.918
Slide the mid-panel	0.861	0.849	0.979	0.968	0.914
AVG	0.893	0.843	0.975	0.962	

**Table 7:** Previous study similarity measurement results for wrist section.

High-Level Activity	Previous Study [19]		This Study	
	Wrist Position	Wrist Twist	Wrist Position	Wrist Twist
Assemble side panel	0.979	0.958	0.979	0.961
Assemble main panel	0.915	0.966	0.971	0.968
Prepare the workspace	0.869	0.948	0.963	0.949
Integrate panel	0.863	0.956	0.981	0.966
Slide the mid-panel	0.908	0.968	0.979	0.968
AVG	0.907	0.959	0.975	0.962

**Table 8:** Similarity measurement results for section B.

High-Level Activity	Neck	Trunk	AVG
Assemble side panel	0.839	0.828	0.834
Assemble main panel	0.819	0.805	0.812
Prepare the workspace	0.806	0.835	0.821
Integrate panel	0.81	0.808	0.809
Slide the mid-panel	0.836	0.83	0.833
AVG	0.82	0.82	

**Table 9:** Similarity measurement results for grand RULA score.

High-Level Activity	Grand Score		
	Right	Left	General
Assemble side panel	0.88	0.884	0.899
Assemble main panel	0.878	0.881	0.898
Prepare the workspace	0.852	0.843	0.861
Integrate panel	0.89	0.882	0.908
Slide the mid-panel	0.869	0.858	0.882
AVG	0.87	0.87	0.89

**Table 10:** *t*-test results for subjects 1–6.

	S1	S2	S3	S4	S5	S6
S1	1.0	0.74 × 10^−3^	1.41 × 10^−15^	2.53 × 10^−7^	9.77 × 10^−13^	1.0
S2	0.74 × 10^−3^	1.0	2.32 × 10^−7^	6.54 × 10^−2^	0.18 × 10^−3^	0.74 × 10^−3^
S3	1.41 × 10^−15^	2.32 × 10^−7^	1.0	0.27 × 10^−3^	2.48 × 10^−2^	1.41 × 10^−15^
S4	2.53 × 10^−7^	6.54 × 10^−2^	0.27 × 10^−3^	1.0	6.32 × 10^−2^	2.53 × 10^−7^
S5	9.77 × 10^−13^	0.18 × 10^−3^	2.48 × 10^−2^	6.32 × 10^−2^	1.0	9.77 × 10^−13^
S6	1.0	0.74 × 10^−3^	1.41 × 10^−15^	2.53 × 10^−7^	9.77 × 10^−13^	1.0
S7	8.53 × 10^−1^	0.22 × 10^−3^	1.04 × 10^−16^	2.76 × 10^−8^	4.06 × 10^−14^	8.53 × 10^−1^
S8	4.36 × 10^−3^	5.11 × 10^−1^	5.05 × 10^−9^	0.99 × 10^−2^	5.55 × 10^−6^	0.44 × 10^−2^
S9	6.30 × 10^−8^	4.57 × 10^−2^	0.24 × 10^−3^	9.24 × 10^−1^	6.87 × 10^−2^	6.30 × 10^−8^
S10	9.07 × 10^−1^	0.32 × 10^−3^	7.12 × 10^−18^	2.59 × 10^−8^	1.25 × 10^−14^	9.07 × 10^−1^
S11	9.79 × 10^−8^	5.49 × 10^−2^	0.19 × 10^−3^	9.48 × 10^−1^	6.90 × 10^−2^	9.79 × 10^−8^
S12	1.01 × 10^−1^	8.66 × 10^−2^	8.12 × 10^−11^	0.43 × 10^−3^	5.85 × 10^−8^	1.01 × 10^−1^

**Table 11:** *t*-test results for subjects 7–12.

	S7	S8	S9	S10	S11	S12
S1	8.53 × 10^−1^	0.44 × 10^−2^	6.30 × 10^−8^	9.07 × 10^−1^	9.79 × 10^−8^	1.01 × 10^−1^
S2	0.22 × 10^−3^	5.11 × 10^−1^	0.46 × 10^−1^	0.32 × 10^−3^	5.49 × 10^−2^	0.87 × 10^−1^
S3	1.04 × 10^−16^	5.05 × 10^−9^	0.24 × 10^−3^	7.12 × 10^−18^	0.19 × 10^−3^	8.12 × 10^−11^
S4	2.76 × 10^−8^	0.99 × 10^−2^	9.24 × 10^−1^	2.59 × 10^−8^	9.48 × 10^−1^	0.43 × 10^−3^
S5	4.06 × 10^−14^	5.55 × 10^−6^	0.69 × 10^−1^	1.25 × 10^−14^	0.69 × 10^−1^	5.85 × 10^−8^
S6	8.53 × 10^−1^	0.436 × 10^−2^	6.30 × 10^−8^	9.07 × 10^−1^	9.79 × 10^−8^	1.01 × 10^−1^
S7	1.0	0.150 × 10^−2^	5.49 × 10^−9^	7.45 × 10^−1^	1.48 × 10^−8^	0.59 × 10^−1^
S8	0.15 × 10^−2^	1.0	0.58 × 10^−2^	0.25 × 10^−2^	0.84 × 10^−2^	2.56 × 10^−1^
S9	5.49 × 10^−9^	0.58 × 10^−2^	1.0	5.06 × 10^−9^	9.78 × 10^−1^	0.19 × 10^−3^
S10	7.45 × 10^−1^	0.25 × 10^−2^	5.06 × 10^−9^	1.0	1.07 × 10^−8^	0.92 × 10^−1^
S11	1.48 × 10^−8^	0.84 × 10^−2^	9.78 × 10^−1^	1.06 × 10^−8^	1.0	0.31 × 10^−3^
S12	5.87 × 10^−2^	2.56 × 10^−1^	0.19 × 10^−3^	0.91 × 10^−1^	0.31 × 10^−3^	1.0

**Table 12:** Number of samples.

High-Level Activity	Number of Samples
Assemble side panel	458
Assemble main panel	1936
Integrate panel	464
Prepare the workspace	1257
Slide the mid-panel	614

## Data Availability

Data available upon request to corresponding author due to ethical restrictions.

## Appendix A

**Table A1:** List of finger joints.

Joint Label	Joint Name
L01–L04	Left thumb joint
L05–L09	Left index finger joint
L010–L14	Left middle finger joint
L15–L19	Left ring finger joint
L20–L24	Left pinky finger joint
L25	Left palm joint
R01–R04	Right thumb joint
R05–R09	Right index finger joint
R10–R14	Right middle finger joint
R15–R19	Right ring finger joint
R20–R24	Right pinky finger joint
R25	Right palm joint

**Table A2:** List of body joints.

Joint Label	Joint Name
01	Head
02	Neck
03	Torso
04	Waist
05	Right hip
06	Left hip
07	Right shoulder
08	Right elbow
09	Right wrist
10	Left shoulder
11	Left elbow
12	Left wrist
13	Right knee
14	Right ankle
15	Left knee
16	Left ankle

**Table A3:** The execution time required to determine the RULA score.

Body Section	All Data (Seconds)	1 Pose (Seconds)
Upper arm	378.3397958	0.00696489
Lower arm	430.0206666	0.007916288
Wrist	461.6637599	0.008498808
Neck	322.8410137	0.005943208
Trunk	297.4668169	0.005476092
Section A (Left)	70.83219051	0.001303956
Section A (Right)	63.70874691	0.00117282
Section A (Max)	76.78008223	0.001413451
Section B	64.08647466	0.001179773
Section C (Left)	56.95984936	0.001048579
Section C (Right)	51.78826308	0.000953375
Section C (Max)	69.78941703	0.001284759
Total	2344.277077	0.043156
